# Whole Transcriptome Analysis of Substantia Nigra in Mice with MPTP-Induced Parkinsonism Bearing Defective Glucocerebrosidase Activity

**DOI:** 10.3390/ijms241512164

**Published:** 2023-07-29

**Authors:** Tatiana Usenko, Anastasia Bezrukova, Margarita M. Rudenok, Katerina Basharova, Maria I. Shadrina, Petr A. Slominsky, Ekaterina Zakharova, Sofya Pchelina

**Affiliations:** 1 Petersburg Nuclear Physics Institute Named by B.P. Konstantinov of National Research Centre «Kurchatov Institute», 188300 Gatchina, Russia; usenko_ts@pnpi.nrcki.ru (T.U.); bezrukova_ai@pnpi.nrcki.ru (A.B.); basharova_ks@pnpi.nrcki.ru (K.B.); pchelina_sn@pnpi.nrcki.ru (S.P.); 2 Department of Molecular Genetic and Nanobiological Technologies, Pavlov First Saint-Petersburg State Medical University, 197022 Saint-Petersburg, Russia; 3 Institute of Molecular Genetics, Russian Academy of Sciences, 123182 Moscow, Russia; rudenok@img.msk.ru (M.M.R.); shadrina@img.ras.ru (M.I.S.); slomin@img.ras.ru (P.A.S.); 4 Research Center for Medical Genetics, Laboratory of Hereditary Metabolic Diseases, 115522 Moscow, Russia

**Keywords:** Parkinson’s disease, transcriptome, 1-methyl-4-phenyl-1,2,3,6-tetrahydropyridine (MPTP), conduritol-β-epoxide (CBE), substantia nigra (SN)

## Abstract

Mutations in the *GBA1* gene represent the major genetic risk factor for Parkinson’s disease (PD). The lysosomal enzyme beta-glucocerebrosidase (GCase) encoded by the *GBA1* gene participates in both the endolysosomal pathway and the immune response. Disruption of these mechanisms is involved in PD pathogenesis. However, molecular mechanisms of PD associated with *GBA1* mutations (GBA-PD) are unknown today in particular due to the partial penetrance of *GBA1* variants in PD. The modifiers of *GBA1* penetrance have not been elucidated. We characterized the transcriptomic profiles of cells from the substantia nigra (SN) of mice with co-injection with 1-methyl-4-phenyl-1,2,3,6-tetrahydropyridine (MPTP) and selective inhibitor of GCase activity (conduritol-β-epoxide, (CBE)) to mimic PD bearing GCase dysfunction (MPTP+CBE), mice treated with MPTP, mice treated with CBE and control mice treated with injection of sodium chloride (NaCl) (vehicle). Differential expression analysis, pathway enrichment analysis, and outlier detection were performed. Functional clustering of differentially represented transcripts revealed more processes associated with the functioning of neurogenesis, inflammation, apoptosis and autophagy in MPTP+CBE and MPTP mice than in vehicle mice, with a more pronounced alteration of autophagy processes in MPTP+CBE mice than in MPTP mice. The PI3K-Akt-mTOR signaling pathway may be considered a potential target for therapy in PD with GCase dysfunction.

## 1. Introduction

Parkinson’s disease (PD) is one of the most common neurodegenerative diseases, and is characterized by the degeneration of dopaminergic neurons (DN) of the midbrain accompanied by protein α-synuclein accumulation [1,2]. Lysosomal enzyme beta-glucocerebrosidase (GCase), encoded by the *GBA1* gene, is significant for the pathogenesis of PD. Mono- and biallelic mutations in the *GBA1* gene have been recognized as the greatest genetic risk factor for PD [3,4,5,6,7]. Homozygous and compound heterozygous *GBA1* mutations result in the most common lysosomal storage disorder (LSD), Gaucher disease (GD), characterized by lysosphingolipid accumulation, presumably in blood macrophages. This is a complex clinical picture that, in the neuronopathic forms of the disease, also includes neurological symptoms [8]. GCase is the lysosomal enzyme involved in ceramide metabolism, and it catalyzes the hydrolysis of glucosylceramide to glucose and ceramide. In its turn, ceramide is involved in neuronal function, immune response, autophagy, etc. [9,10,11].

Despite previous data demonstrating that PD associated with mutations in *GBA1* gene (GBA-PD) is associated with the impairment of processes encompassing the endo-lysosomal pathways, vesicular trafficking, lipid metabolism, and the cell stress response, not every carrier of *GBA1* mutations develops PD during their lifetime. Our previous study based on the comparative transcriptome analysis revealed a pronounced alteration of autophagy and immune response in GBA-PD compared to non-manifesting *GBA1* mutation carriers in peripheral blood monocyte-derived macrophages [12]. Additionally, we, and others, have previously demonstrated that mutations in the *GBA1* gene lead to a decrease in GCase activity in the blood in *GBA1* mutation carriers independent of PD status [13,14,15,16,17]. Therefore, it is important to determine the additional modifiers that are responsible for the onset of PD in some *GBA1* mutation carriers.

Mice models based on the administration of MPTP (1-methyl-4- phenyl-1,2,3,6-tetrahydropyridine), which became the first neurotoxin used to mimic PD, are the most frequently used models recapitulating PD-like symptoms [18,19,20,21]. Conduritol-β-epoxide (CBE) is used for inhibition of GCase activity due to its forming covalent bonds with the catalytic site of the enzyme, causing accumulation of the GCase substrates glucosylceramide and glucosylsphingosine. CBE has long been used to inhibit GCase activity in an attempt to replicate GD-like features in rodents [22]. Recently, Mus and colleagues first demonstrated the possibility of using CBE with MPTP in creation of a PD mice model with partial deficiency of GCase activity [23]. We have replicated the double toxic model (MPTP+CBE) using the presymptomatic PD mouse model described earlier [24] and combined a low dosage of MPTP (12 mg/kg, i.p. for 14 days) with a single CBE injection (100 mg/kg) [25].

Here, we first generated the transcriptomic profiles of substantia nigra (SN) cells for mice with coadministration of MPTP and CBE as described earlier [25], mice with administration of MPTP alone, CBE alone, and sodium chloride (NaCL) as a vehicle. We identified new patterns of change in gene expression profile, as well as processes that may be associated with the initiation of PD development in the presence of GCase defects. We also conducted a comparative analysis of the transcriptomic dataset of differentially expressed genes (DEGs) obtained by us in the current study and earlier during the analysis of the transcriptome of the primary culture of macrophages derived from peripheral blood lymphocytes of patients with GBA-PD and controls [12].

Herein, abnormalities in the autophagy process, in particular in the PI3K-Akt-mTOR signaling pathway in MPTP+CBE mice, were demonstrated, suggesting that possible modifiers of the mammalian target of rapamycin (mTOR) may be suitable for targeted therapeutic interventions.

## 2. Results

### 2.1. Changes in the Transcriptome Attributed to the Dysfunction of GCase

**Figure 1:**
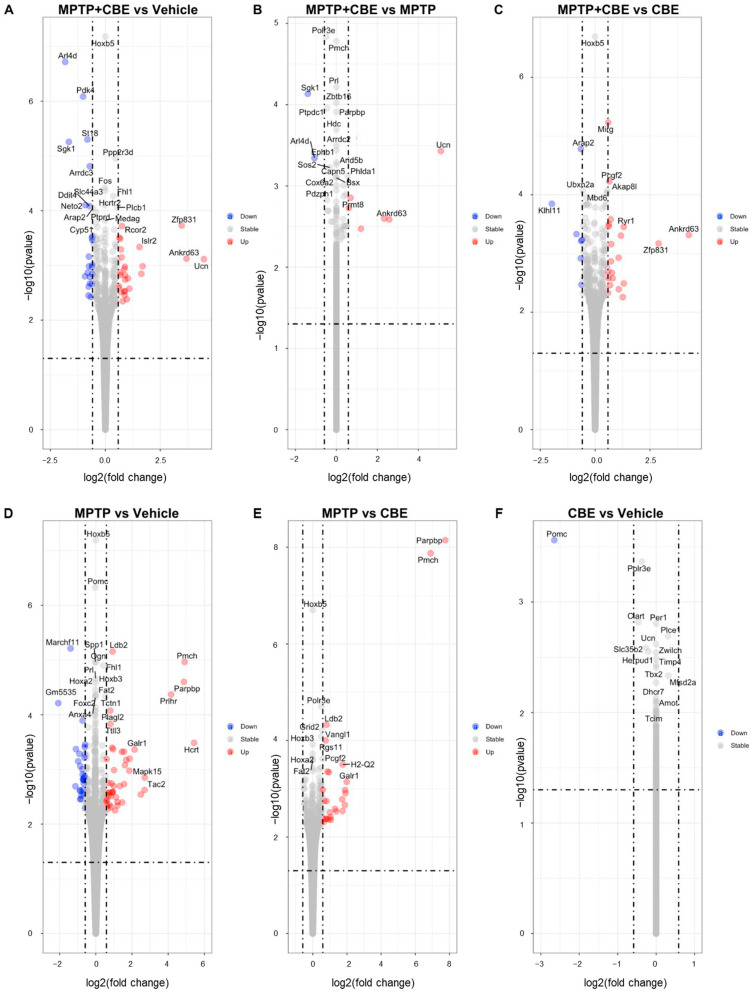
Volcano plot for DEGs between the studied groups (false discovery rate (FDR) < 0.05 and |fold change (FC)| > 1.5); the upregulated genes are represented by red dots and the downregulated genes are represented by blue dots. (**A**) MPTP+CBE vs. vehicle, (**B**) MPTP+CBE vs. MPTP, (**C**) MPTP+CBE vs. CBE, (**D**) MPTP vs. vehicle, (**E**) MPTP vs. CBE, (**F**) CBE vs. vehicle.

Pairwise comparison of data from the groups of mice with MPTP vs. NaCl (vehicle mice), mice with MPTP vs. CBE, mice with MPTP vs. MPTP+CBE, mice with MPTP+CBE vs. CBE, mice with MPTP+CBE vs. vehicle mice and mice with CBE vs. vehicle mice was made using the DESeq library in R. Analysis revealed the expression of genes whose levels differed by more than 1.5 times, with a *p*-value < 0.01 between compared groups. We identified 64 DEGs in SN from mice with MPTP vs. vehicle mice (40 upregulated DEGs, 24 downregulated DEGs), 23 DEGs in mice with MPTP vs. CBE (22 upregulated DEGs, 1 downregulated DEG), 6 upregulated DEGs and 2 downregulated DEGs in mice with MPTP+CBE vs. MPTP, 23 DEGs in mice with MPTP+CBE vs. CBE (16 upregulated DEGs, 7 downregulated DEGs), 37 DEGs in mice with MPTP+CBE vs. vehicle mice (21 upregulated DEGs, 16 downregulated DEGs) and 1 downregulated DEG in mice with CBE vs. vehicle mice (Figure 1, Tables S1–S6).

**Figure 2:**
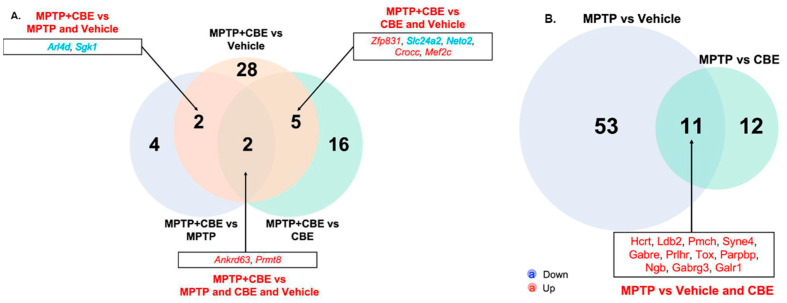
Venn Diagram of DEGs between the three studied groups of mice with (**A**) MPTP+CBE vs. CBE, MPTP+CBE vs. vehicle, MPTP+CBE vs. MPTP; (**B**) MPTP vs. vehicle, MPTP vs. CBE.

Following the results of the Venn Diagram, we found common alterations of gene expression between the groups (Table S7). Mice with MPTP vs. mice with CBE and vs. vehicle mice were characterized by increased expression of *Hcrt*, *Pmch*, *Gabre*, *Prlhr*, *Tox*, *Parpbp*, *Ngb*, *Gabrg3*, and *Galr1*. Administration of MPTP led to a decrease in *Armh4*, *Hsd17b7* expression and an increase in *Islr2*, *Ttll3*, *Aldh3b2*, *Drc1* in groups of mice with MPTP vs. vehicle mice and MPTP+CBE vs. vehicle mice. Elevated *Prmt8* and *Anrkd63* expression levels in mice with MPTP+CBE vs. CBE, vs. MPTP, vs. vehicle mice was found. In groups of mice with MPTP+CBE vs. MPTP and vs. vehicle mice, a decreased expression of *Sgk1* and *Arl4d* genes was observed, and in groups of mice with MPTP+CBE vs. CBE and vs. vehicle mice, two downregulated genes (*Slc24a2*, *Neto2*) and three upregulated genes (*Mef2c*, *Zfp831*, *Crocc*) were observed (Figure 2A). In groups of mice with MPTP vs. vehicle and mice with MPTP vs. mice with CBE, 11 common DEGs were found (*Hcrt*, *Pmch*, *Gabre*, *Prlhr*, *Tox*, *Parpbp*, *Ngb*, *Gabrg3*, *Galr1*) (Table S7, Figure 2B).

### 2.2. Gene Expression Outliers Highlight Targeted Pathways in Cojoined Influence of MPTP and CBE

**Figure 3:**
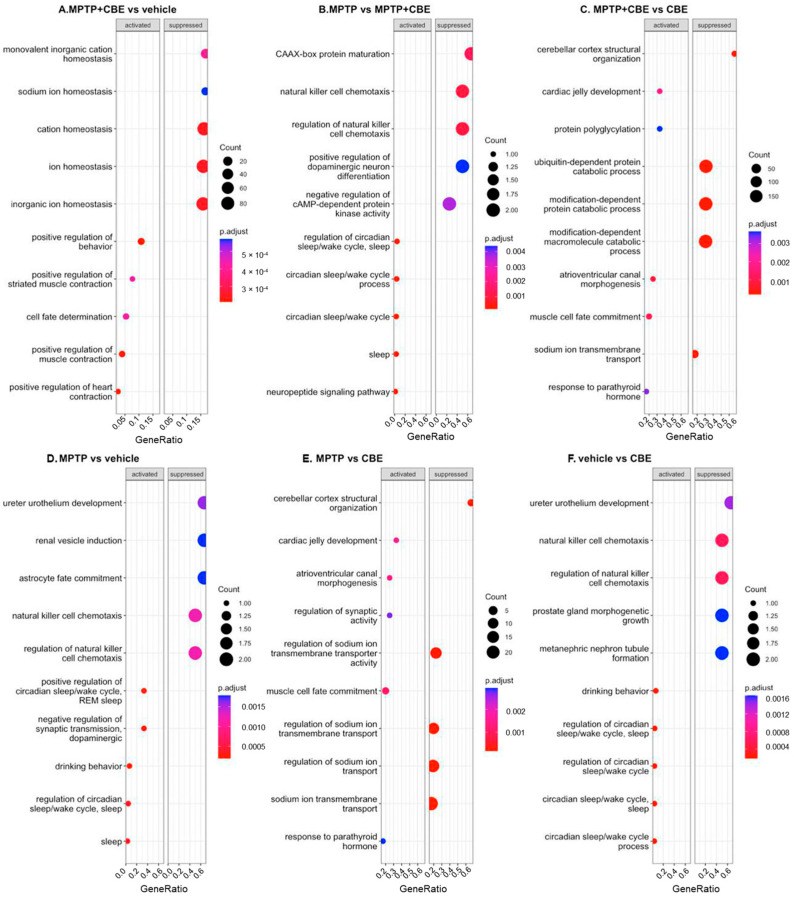
Enriched Biological Process GO terms: dot plot. The 10 GO processes with the largest gene ratios are plotted in order of gene ratio. (**A**) MPTP+CBE mice vs. vehicle, (**B**) MPTP+CBE mice vs. CBE mice, (**C**) MPTP mice vs. MPTP+CBE mice, (**D**) MPTP+CBE mice vs. vehicle, (**E**) MPTP+CBE mice vs. CBE mice, (**F**) MPTP mice vs. MPTP+CBE mice. The size of the dots represents the number of DEGs associated with the GO term, and the color of the dots represent the P-adjusted values.

**Figure 4:**
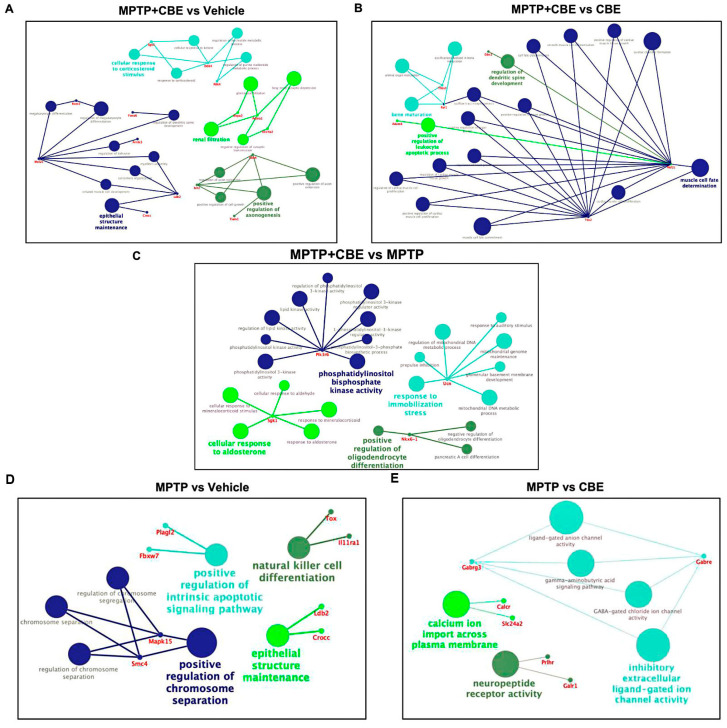
Networks of selected metabolic processes and DEGs in (**A**) MPTP+CBE mice vs. vehicle, (**B**) MPTP+CBE mice vs. CBE mice, (**C**) MPTP+CBE mice vs. MPTP mice, (**D**) MPTP mice vs. vehicle, (**E**) MPTP mice vs. CBE mice (obtained using CluePedia v. 1.5.9 + ClueGo v. 2.5.9).

**Figure 5:**
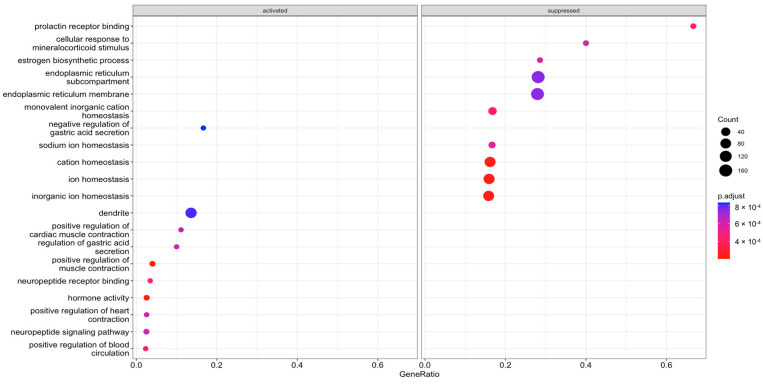
All enriched GO terms: dot plot. The 20 GO processes with the largest gene ratios are plotted in order of gene ratio in MPTP+CBE mice vs. vehicle. The size of the dots represents the number of DEGs associated with the GO term, and the color of the dots represent the P-adjusted values.

Gene Ontology (GO) term enrichment analysis and gene set enrichment analysis (GSEA) were conducted for all determined DEGs. We considered “biological process” GO terms as well as all types of GO terms together (“biological process”, “molecular function”, “cellular component”) with a *p*-value (Bonferroni corrected) <0.05. All pathways determined with GSEA are shown in Figure 3. All pathways determined using GO analysis are shown in Figure 4. Using GSEA analysis, we found downregulated pathways associated with ion metabolism and upregulated pathways associated with neuronal function in MPTP+CBE mice vs. vehicle, upregulated pathways associated with inflammation and downregulated pathways associated with neuronal function in MPTP+CBE mice vs. MPTP mice and downregulated pathways associated with ion metabolism in MPTP+CBE mice vs. CBE mice (Figure 3). The alterations in pathways associated with neuronal function and inflammation were found using GO analysis in MPTP+CBE mice vs. vehicle and in MPTP+CBE mice vs. CBE and inflammation in MPTP+CBE vs. MPTP mice (Figure 4). Pronounced suppression of pathways associated with the endoplasmic reticulum (ER) was found in MPTP+CBE mice vs. vehicle using GSEA analysis with all types of GO terms (Figure 5). Mice with MPTP were characterized by pronounced alteration of ion metabolism and neuronal function compared to vehicle mice, and mice with MPTP were characterized by the disruption of apoptotic and inflammation pathways compared to CBE mice (Figure 3 and Figure 4).

### 2.3. Overlapping Analysis of Enriched Pathways in MPTP-CBE Mice Model and Data Set of RNA-Seq Peripheral Blood Monocyte-Derived Macrophages from L444P/N GBA-PD Patients

Raw data from our previous study comprised RNA-seq of peripheral blood monocyte-derived macrophages from L444P/N GBA-PD patients, asymptomatic *GBA1* mutation carriers (GBA carriers) and controls that had been deposited in NCBI’s Gene Expression Omnibus [26] and are accessible through GEO Series accession number GSE184956 (https://www.ncbi.nlm.nih.gov/geo/query/acc.cgi?acc=GSE184956 (accessed on 9 September 2021)). DEGs of compared groups are presented in Supplementary Tables S2–S4 of our previous article [12].

**Figure 6:**
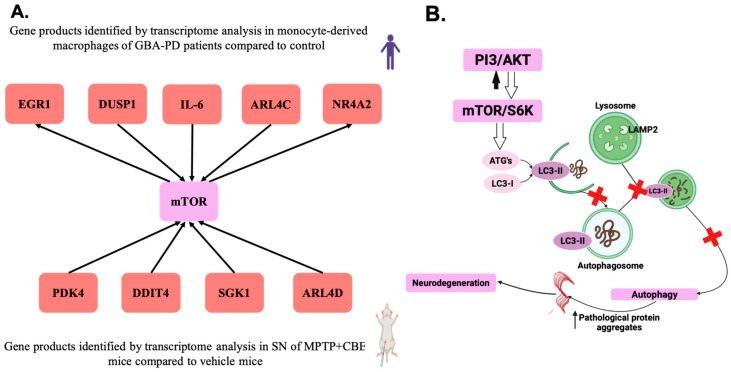
PI3K-Akt-mTOR pathway. (**A**) DEGs identified during the analysis of the top 10 DEGs in two datasets: monocyte-derived macrophages from patients with L444P/N GBA-PD compared to control and SNs MPTP+CBE mice and vehicle; (**B**) Schematic representation of the role of the PI3K-Akt-mTOR pathway in neurodegeneration—the mTOR complex mTORC1 acts upstream of the autophagic pathway to suppress autophagic membrane formation. Alteration of mTOR activity leads to disruption of autophagosome formation due to the suppressed kinase activity of cytosolic Atg proteins and, as a consequence, impairment of conjugation of LC3-I to PE for formation of LC3-II. LC3-II proteins then specifically associate with a newly formed crescent-shaped membrane termed a phagophore. Next, the phagophore is unable to elongate around cytosolic contents until the contents are completely sequestered within a fully formed double-membraned autophagosome. The mature autophagosomes cannot become acidic and fuse with lysosomes to form the degradative autolysosome. There is a violation of the process of autophagy and, as a result, a violation of the degradation of proteins, in particular, the alpha-synuclein protein, which possibly leads to neurodegeneration.

Next, we focused on top genes determined in the DEGs dataset of mice with MPTP+CBE vs. vehicle mice and the DEGs dataset of macrophages of L444P/N GBA-PD patients vs. controls [12]. Based on the literature and the website GeneCards (https://www.genecards.org, accessed on 1 June 2023), the products of the most of top genes of the two datasets are involved in the PI3K-Akt-mTOR pathway (Figure 6, Tables S8 and S9). Moreover, the genes from one ARL4 family (ADP ribosylation factor like GTPase 4) were present in the top list of the two datasets (*Arl4d*, *ARL4C*).

## 3. Discussion

The whole transcriptome analysis of cells from the SN of a mouse model of PD bearing defective GCase activity (double neurotoxic MPTP+CBE model) compared to vehicle mice was conducted for the first time. DEGs of CBE-treated and MPTP-treated mice were also analyzed.

In MPTP-treated animals, we revealed the disruption of pathways associated with neuronal function, apoptosis, vesicular transport and immune response (Figure 3 and Figure 4). Thus, in some ways, we replicated the analysis conducted by Alieva and colleagues, who studied the transcriptome profile of SN in the MPTP-induced early stage of PD [27,28]. These authors found a dysregulation of pathways involved in vesicular transport and also in mitochondrial function, apoptosis, ubiquitin-dependent proteolysis, RNA splicing and myelination.

As for CBE-treated mice, Vardi and coauthors previously used CBE for the development of symptoms associated with neurological forms of GD and remarked on the similarities in the gene expression profiles in brain samples of CBE-treated mice and a genetic GD mouse model (Gba^flox/flox^; nestin-Cre mice) [28]. The authors suggested that CBE injection may provide a rapid and relatively easy way to induce symptoms typical of neuronal forms of GD [29]. A recent study found an alteration of expression in genes involved in the IFN response in brain samples of CBE-treated mice [30]. These data supported a previous study that demonstrated an activation of inflammation processes in liver and lung samples of *Gba1* point-mutated mice (V394L/V394L and D409V/null) [31]. Additionally, a recent study based on single cell transcriptome analysis of brain samples of GD model mice (*Gba*^lnl/lnl^ mice with 134 germ-line deletion of *Gba*) found activation of neuroinflammation in the form of attrition of homeostatic microglia, emergence of DAM, influx of CCR2+ MFs, activation of the ISG pathway and infiltration of activated NK cells [32]. We found one downregulated DEG, *Pomc*, in CBE-treated mice (Table S6). The product of this gene, the precursor protein proopiomelanocortin, plays multiple roles in the cell, such as stress response, immune system, the central melanocortin system and regulating feeding behavior [33]. GSEA analysis revealed the alteration of pathways associated with inflammation (Figure 3). Downregulated expression of *Pomc* may be associated with inflammation in response to CBE injection.

Our previous study performed unbiased transcriptomic analysis of monocyte-derived macrophages comparing GBA-PD and non-manifesting GBA carriers and control subjects [12]. We found an aberration of immune response, neuronal function and zinc metabolism pathways in GBA-PD and GBA carriers and more pronounced altered expression of genes involved in autophagy in GBA-PD patients than in GBA carriers [12]. Another study, using gene-based outlier analysis, found the disruption of lysosomal, membrane trafficking, and mitochondrial processing in circulating monocytes CD14+ of GBA-PD patients compared to GBA carriers [34].

Here, we focused on the analysis of MPTP+CBE-treated mice vs. vehicle mice. We revealed 8 DEGs in mice with MPTP+CBE vs. MPTP (6 upregulated DEGs, 2 downregulated DEGs), 23 DEGs in mice with MPTP+CBE vs. CBE (16 upregulated DEGs, 7 downregulated DEGs) and 37 DEGs in mice with MPTP+CBE vs. vehicle mice (21 upregulated DEGs, 16 downregulated DEGs). Following the GSEA analysis, we found upregulated pathways associated with neuronal processes and downregulated pathways associated with ion metabolism. Following the GO analysis, the alteration of inflammation processes was found (Figure 3 and Figure 4). The revealed upregulated neuronal activity may be associated with induced processes of neurodegeneration, which is supported by a decrease in dopamine level in the striatum of mice with MPTP+CBE compared to vehicle mice [25]. The disruption of ion metabolism may be also associated with the involvement of several ion channels in the release of dopamine in SN neurons. An earlier study reported that ion channels play a central role in driving the high vulnerability of dopaminergic neurons to degeneration during PD [35]. Dysregulation of ion channels causes the aberrant movement of various ions in the intracellular milieu, which leads to the disruption of intracellular signaling cascades, alterations in cellular homeostasis, and bioenergetic deficits [35,36]. It is well known that *GBA1* mutations lead to increased secretion of proinflammatory cytokines. In our previous study, we reported increased proinflammatory cytokines in the plasma of GBA-PD patients [37]. Mice with p.Asp409Val/knockout in *GBA1* had increased levels of inflammatory cells and cytokines including IFN*γ*, TNF, IL-1*β*, IL-6, and IL-17A/F, as well [38]. Another study demonstrated that glucosylceramide, one of the main substrates of GCase, can activate myeloid cells and increase the levels of inflammatory cytokines in the thymus of hematopoietic-specific GBA1-deficient mice [39].

Enriched all GO terms analysis additionally revealed pronounced suppression of ER pathways in MPTP+CBE mice (Figure 5). ER plays a key role in the synthesis, glycosylation and folding of proteins [40], and ER stress leads to the accumulation of unfolded or misfolded proteins. Several reviews have discussed how ER stress is a causative factor in PD [41,42]. *GBA1* mutations may lead to the production of a misfolded protein, which can be retained in the ER to induce ER stress [43]. As Maor and colleagues demonstrated, *GBA1* mutations lead to their retention in the ER and subsequent activation of the UPR (unfolded protein response) in the Drosophila model [44]. Post-mortem analysis of brains of Lewy bodies dementia (LDB) patients carrying *GBA1* mutations showed an abnormal UPR response that was associated with ER stress [45]. All of these observations in a model with GCase dysfunction were accompanied by an increased α-synuclein level that proved the link between ER stress and α-synuclein metabolism [46,47,48]. Our mice model with MPTP+CBE was also characterized by an increased α-synuclein level in the striatum [25], along with downregulated ER activity followed by GSEA analysis (Figure 5), which supports previous results. Kuo and colleagues suggested that GCase that fails to fold in the ER is efficiently targeted to lysosomes by chaperone-mediated autophagy (CMA), but blocks the multimerization of LAMP2A, resulting in a disruption of proteostasis and α-synuclein accumulation [49]. In our previous study, we found a decrease in expression of the *LAMP2* gene in CD45+ blood cells in GBA-PD and PD patients compared to controls, with a more pronounced decrease in *LAMP2* expression in GBA-PD, supporting the role of the disruption of the autophagy–lysosome pathway in GCase dysfunction [50].

Next, we focused on the top ten DEGs in comparative groups of MPTP-CBE mice vs. vehicle mice and compared them with the top ten from the dataset of DEGs revealed during our previous research conducted on peripheral blood monocyte-derived macrophages from L444P/N GBA-PD patients [12]. The products of the top DEGs in MPTP+CBE vs. vehicle mice appeared to be involved in the PI3K-Akt-mTOR pathway (Figure 6). It is interesting that similar alterations of this pathways were determined in peripheral blood monocyte-derived macrophages from L444P/N GBA-PD patients (Figure 6). The PI3K/AKT/mTOR pathway regulates autophagy, apoptosis, cell cycle, inflammation, and according to the previous data, may be involved in neurodegeneration [51,52].

In our current study, MPTP+CBE mice were characterized by decreased expression of *Sgk1, Pdk4, Arl4d, Arrdc3,* and *Ddit4*. The *Sgk1* gene encodes serum- and glucocorticoid-dependent kinase 1 (SGK1). Earlier, a downregulated level of Sgk1 was reported in SNs of mice with chronic MPTP intoxication [53]. Pyruvate dehydrogenase kinase (PDK) is also located in the outer mitochondrial membrane and can negatively regulate PDH activity by phosphorylating one of its subunits. Early overexpression of PDK4 may protect cells from damage caused by ROS, and attenuate neuronal apoptosis by reducing oxidative stress [54,55]. The PI3K/AKT/mTOR pathway affects the oxidative stress pathway through other downstream signaling molecules, such as FoxO3a, in addition to GSK-3beta, to influence PD [56]. We found an increased expression of Foxo6 in MPTP+CBE mice (Figure 1, Table S2). Members of the FoxO subfamily shuttle from the cytoplasm to the nucleus and play an important role in cell proliferation, apoptosis, differentiation and oxidative stress resistance. It was hypothesized that SGK1 may play a critical role downstream of PDK1 in sustaining mTORC1 activity [57]. mTORC1 activity was augmented with PDK4 overexpression and reduced by PDK4 suppression in various cell lines [58]. The coding product of the *DDIT4* gene is a stress-induced protein called RTP801/REDD1 [59]. RTP801 is a negative regulator of mTOR [60,61]. RTP801/REDD1 inhibits the activity of mTOR and participates in the regulation of diverse cell functions including proliferation, apoptosis and differentiation. RTP801 was elevated in the SN of PD patients [61]. Here, we found downregulated *Ddit4* expression in MPTP+CBE mice vs. vehicle, which may by associated with activation of mTOR activity. A recent study also demonstrated decreased *Ddit4* in a mouse model with chronic MPTP treatment after 14 days compared to vehicle [62]. ARL4D, ARL4A and ARL4C are closely related members of the ADP-ribosylation factor/ARF-like protein (ARF/ARL) family of GTPases. ARL4D is located primarily at the plasma membrane, but can also be detected in the nucleus and cytoplasm. Dysfunctional GTP-binding-defective ARL4D is targeted to mitochondria functions, vesicular transport [63]. Interestingly, common family genes were found when comparing the two data sets (peripheral blood mononuclear cells (PBMC)-derived macrophages and SN of mice). There were two genes (*ARL4C* and *Alr4d*) encoding the proteins from Arl4 family (ADP ribosylation factor (Arf)-like 4 proteins). *ARL4C* and *Alr4d* are involved in the PI3K/AKT/mTOR pathway [64].

When comparing datasets of DEGs in MPTP+CBE mice to MPTP mice, CBE mice and vehicle mice, two DEGs (*Ankrd63*, a gene with products without well-established function, and *Prmt8*, which regulates the maturation of synapses and neural circuits during brain development [65]) were identified. As expected, five common DEGs in MPTP+CBE mice compared to CBE mice and vehicle mice were involved in neurogenesis and dopamine transport (*Zfp831*, *Slc24a2*, *Neto2*, *Crocc*, *Mef2c*), thus suggesting a potentiating effect of MPTP on alteration of neuronal function (Figure 2). Two common DEGs (*Arl4d*, *Sgk1*) were identified in MPTP+CBE mice compared to MPTP mice and vehicle mice. They are involved in the PI3K-Akt-mTOR pathway, thus suggesting a potentiating effect of CBE and, as a consequence of GCase dysfunction, an effect on the disruption of autophagy processes (Figure 2). Interestingly, monocyte-derived macrophages of patients with L444P/N GBA-PD were also characterized by a more pronounced change in the genes involved in the PI3K-Akt-mTOR pathway compared to L444P/N GBA carriers and controls (*DUSP1*, *ARL4C*) (Table S9) [12].

We found a similar alteration in the PI3K-Akt-mTOR pathway in condition of GCase dysfunction in our PD models, both in primary macrophages from patients with GBA-PD and in the double toxic model MPTP+CBE-treated mice (Figure 6B). Recently, disturbances in the autophagy–lysosomal pathway, which co-occur with upstream perturbations in mTOR activation, were found using proteome analysis of induced pluripotent stem cell (iPSC) dopamine neurons of GBA-PD patients [66]. Additionally, single-cell RNA sequencing and proteomics of brain samples from GBA-PD patients confirmed reduced CMA activity and proteome changes comparable to those found in brain samples from heterozygous L444P/N *GBA1* mice [49]. Interestingly, glycoproteome analysis revealed a number of significantly enriched pathways, including ceramide catabolic processes, and also an increased level of glycosylated LAMP1, LAMP2 and cathepsin D, which are necessary for transport from the ER via the Golgi to the lysosome, in GBA-PD iPSC-dopamine neurons. Increased phosphorylated mTOR level was supported with Western blot analysis [66]. Earlier, we found differences in lysosomal hydrolase activity (alpha-galactosidase (GLA), alpha-iduronidase (IDUA)) in iPCS neurons of GBA-PD compared to GBA carriers, suggesting that a more pronounced imbalance of sphingolipid metabolism may lead to impaired lysosomal clearance and launch the diseases associated with GCase deficiency [67]. Moreover, treatment with a compound (Genz-123346) that inhibits glycosphingolipid biosynthesis decreases mTOR activity and restores TFEB expression in GBA-PD iPSC neurons, demonstrating a possible link between mTOR-TFEB alterations and lipid accumulation [68]. Inhibition of mTOR by selective inhibitors restored TFEB activity, which plays a role in the regulation of lysosomal biogenesis and autophagy, decreased ER stress and reduced alpha-synuclein protein level, suggesting the improvement of neuronal protiostasis on GBA-PD iPSC neurons [68]. However, it is still necessary to look for what distinguishes between GBA-PD and GBA carriers, as the penetrance of heterozygous *GBA1* mutations for PD is variable from 10% to 30% [69].

Taken together, our data support dysfunction of the autophagy–lysosomal pathway as a central pathogenic event in GBA-associated neurodegenerative disease, as has been suggested [70,71]. Although autophagic disturbances were also revealed in MPTP-treated mice with targeted or whole transcriptomic approaches including our previous study [72,73,74,75], our findings demonstrated a more pronounced change in the PI3K-Akt-mTOR pathway in the case of GCase deficiency.

The current study has some limitations. The small size of the studied groups of mice may influence the outcome of differential expression analysis for genes with small differences in expression levels, eliminating nonspecific gene expression differences.

## 4. Materials and Methods

### 4.1. Brain Samples from Mice

Sixteen mice C57BL/6 8–12 weeks old weighing 22–26 g were generated and separated into four groups with four animals in each, and treated with the following solutions: 0.9% sodium chloride solution (vehicle mice), conduritol β-epoxide (CBE) with MPTP (MPTP-CBE), CBE, MPTP. The animals were maintained at 21–23 °C in a 12 h light/dark cycle having free access to food and tap water. In the CBE group, CBE was intraperitoneally injected once in an individual dose of 100 mg/kg. In the MPTP group, MPTP was subcutaneously injected twice, with 2 h intervals between the injections, at the individual dose of 12 mg/kg, as described earlier by Ugrumov et al. [24]. In the MPTP+CBE group, MPTP was subcutaneously injected twice with 2 h intervals between the injections at the individual dose of 12 mg/kg, and CBE was injected intraperitoneally once with an individual dose of 100 mg/kg simultaneously with the second injection of MPTP. To assess motor dysfunction, the “Grip strength” (GRIP-test) [76] and “Open field” behavioral tests [77] were carried out 2 weeks after injections. Animals were scarified 2 weeks after injection day. The brain samples were removed from the skull and cut along the midsagittal plane. The substantia nigra was dissected under a dissecting microscope with an ocular micrometer (Nikon SMZ660, Nikon, Melville, NY, USA). Samples of peripheral blood were obtained from all animals. SN samples were obtained and frozen and kept at −70 °C until RNA isolation.

### 4.2. RNA Isolation and RNA Sequencing (RNA-Seq)

Whole-transcriptome analysis was performed using pooled brain tissues of SN of mice with MPTP-CBE-induced PD with GCase dysfunction, MPTP-induced PD, CBE and control mice (vehicle) with NaCl. For this purpose, 5 mg of brain tissue was taken from each of the four animals in each group. Total RNA was extracted from brain tissue with Trisol reagent and PureLink RNA micro Kit (PureLink RNA micro Kit, Invitrogen, CA, USA), according to the manufacturer’s instruction. The quality was checked with a BioAnalyser (2100 Bioanalyzer Instrument, Agilent, CA, USA) and RNA 6000 Nano Kit (RNA 6000 Nano Kit, Agilent, CA, USA). PolyA RNA was purified with Dynabeads^®^ mRNA Purification Kit (Dynabeads^®^ mRNA Purification Kit, Ambion, TX, USA). The Illumina library was made from polyA NEBNext^®^ Ultra™ II RNA Library Prep (NEBNext^®^ Ultra™ II RNA Library Prep, NEB, MA, USA), according to the manual. Sequencing was performed on a HiSeq1500 (Illumina, CA, USA) with 50 bp read length. At least ten million reads were generated for each sample.

### 4.3. Quality Control

Quality control for each sample was performed using FastQC (v0.11.9) [78] and RSeQC (v4.0.0) [79]. In this step, clean data (clean reads) were obtained by removing low-quality reads, reads containing adapters, and reads containing ploy-N from raw data. The removal adapter was conducted with Cutadapt [80]. All downstream analyses were based on clean data.

### 4.4. Reads Mapping to Reference Genome

Mouse reference genome assembly GRCm39 and gene model annotation files were downloaded from the Gencode website (https://www.gencodegenes.org/mouse/ (accessed on 28 March 2023)) directly (release M32). HISAT2 (v2.2.1) [81] was used with default parameters to build the index of the reference genome and to map reads to the genome.

### 4.5. Quantification of Gene Expression Level

Counting of sequencing reads mapping to each gene after the alignment step was performed using the HTSeq-count function from the HTSeq framework (v.0.6.1) [82].

### 4.6. Analysis of Gene Differential Expression

Gene differential expression analyses of the three groups were performed using the DESeq2 package (v.1.30.1) [83] in R (v.4.1.2). DESeq2 provides statistical routines for determining differential expression in digital gene expression data using a model based on negative binomial distribution. The resulting *p*-values were adjusted using Benjamini and Hochberg’s approach for controlling the false discovery rate (FDR). The groups of mice were subdivided into subgroups based on the symptomatic (mice with MPTP vs. NaCl (vehicle mice)) and GCase dysfunction (mice with MPTP-CBE, mice with CBE and mice without GCase dysfunction (MPTP, NaCl/vehicle). Results from the comparison of each pair of groups were then extracted. A threshold of FDR < 0.05 was utilized as the threshold of significance. Detected differential expression of genes was considered statistically significant at *p*-value ≤ 0.01 and a fold change (FC) threshold > 1.5. The differentially expressed genes were visualized in a volcano plot built using ggplot (v.3.3.3) in R (v 4.0.3).

### 4.7. GO Enrichment Analysis of Differentially Expressed Genes

GO enrichment analysis of differentially expressed genes was performed using the GO resource (http://geneontology.org (accessed on March 2023)) and was carried out using the apps ClueGO v. 2.5.7 [84] and CluePedia v. 1.5.3 [85] for Cytoscape v. 3.6.1. GO terms with a corrected *p*-value of less than 0.05 were considered. Term groups were selected using ClueGO based on the number of common genes/terms (>50%). Term clusters were selected based on common genes. Functional clustering and annotation of selected genes were performed using the STRING database (version 10.0). Venn diagrams were constructed in the VennDiagram software package for R (version 3.4.0). A corrected *p* value (Benjamini) was calculated for each functional cluster.

## 5. Conclusions

We identified a set of genes and molecular pathways that are specific to PD with GCase dysfunction in the SN of mice treated with co-injection of MPTP and CBE. These genes and pathways related to dysregulation of lysosomal, membrane trafficking, inflammation and mitochondrial processes, suggesting that alteration of these processes is more pronounced in samples manifesting GBA-PD. We found a common altered PI3K-Akt-mTOR signaling pathway that regulates key process in GBA-PD pathogenesis, such as autophagy and immune response based on comparative analysis of transcriptomic data of human monocyte-derived macrophages and SN cells of mice with PD and GCase dysfunction. Further investigation will clarify the possible role as PD biomarkers of these hits.

## Data Availability

The data discussed in this publication have been deposited at ArrayExpress database at EMBL-EBI (https://www.ebi.ac.uk/biostudies/ (accessed on 25 July 2023)) under accession number S-BSST1159 https://www.ebi.ac.uk/biostudies/studies/S-BSST1159 (accessed on 25 July 2023) for array design “S-BSST1159”.

## Supplementary Materials

The following supporting information can be downloaded at: https://www.mdpi.com/article/10.3390/ijms241512164/s1. References [64,86,87,88,89,90,91,92,93] are cited in the supplementary materials.
