# Supplementary material for: Whole Transcriptome Analysis of Substantia Nigra in Mice with MPTP-Induced Parkinsonism Bearing Defective Glucocerebrosidase Activity
Source: Int J Mol Sci. 2023 Jul 29;24(15):12164. doi: 10.3390/ijms241512164 (PMC10418497; doi:10.3390/ijms241512164)
Supplement: Supplementary file 1 [file ijms-24-12164-s001.zip › ijms-2506134-supplementary.pdf]

Table S1. Differentially expressed genes in SN from mice with MPTP+CBE compared to mice with CBE

DEGs	p-value	Log2FoldChange
Mirg	$5.87*10^{-6}$	0.59399156
Arap2	$1.66*10^{-5}$	-0.64772314
Pcgf2	$5.94*10^{-5}$	0.64560150
Klhl11	$1.42*10^{-4}$	-1.97081617
Dbn1	$3.47*10^{-4}$	0.61752537
Ryr1	$3.52*10^{-4}$	1.30517771
Slc24a2	$4.68*10^{-4}$	-0.84869852
Ankrd63	$4.84*10^{-4}$	4.26887775
Tbx2	$4.97*10^{-4}$	1.17503584
Zc3h12c	$5.89*10^{-4}$	-0.60031129
Kif5b	$6.15*10^{-4}$	-0.63478363
Zfp831	$6.75*10^{-4}$	2.89086435
Atp13a5	$6.95*10^{-4}$	0.73688808
Mef2c	0.00118040	1.07138316
Neto2	0.00121295	-0.65772502
Syne4	0.00136080	0.72538958
Crocc	0.00213178	0.76535787
Adam8	0.00264611	0.80238999
Prmt8	0.00264718	0.60765600
Cdk3	0.00323735	1.30858203
Hecw1	0.00341241	-0.62433392
Tnfrsf25	0.00406376	1.07777881
Thbs3	0.00469813	0.58576518

SN – substantia nigra; MPTP - 1-methyl-4-phenyl-1,2,3,6-tetrahydropyridine; CBE - conduritol B epoxide; DEGs - differentially expressed genes

Table S2. Differentially expressed genes in SN from mice with MPTP+CBE compared to mice with vehicle

DEGs	p-value	Log2FoldChange
Arl4d	$1.91*10^{-7}$	-1.83005951
Pdk4	$8.24*10^{-7}$	-1.01097351
St18	$5.00*10^{-6}$	-0.81759584
Sgk1	$5.53*10^{-6}$	-1.66604096
Arrdc3	$1.54*10^{-5}$	-0.70916389
Neto2	$7.91*10^{-5}$	-0.91135940
Ddit4	$8.26*10^{-5}$	-0.72050667
Rcor2	$1.91*10^{-4}$	0.75428811
Zfp831	$1.88*10^{-4}$	3.47671204
Ciart	$2.93*10^{-4}$	-0.58955761
Idi1	$3.14*10^{-4}$	-0.61682728
Ldb2	$3.26*10^{-4}$	0.67938838
Tlcd4	$3.51*10^{-4}$	-0.59426052
Islr2	$4.61*10^{-4}$	1.55549782
Ttll3	$5.13*10^{-4}$	0.70661232
Ankrd63	$7.55*10^{-4}$	3.69671419
Lrrn1	$9.82*10^{-4}$	-0.60061231
Npy2r	0.00103964	1.70016592
Slc24a2	0.00104371	-0.74572326
Nsun7	0.00105934	0.89010552
Rhpn2	0.00108437	-0.60907209
Crocc	0.00115782	0.86478225
Tiam1	0.00134855	0.66832341
Aldh3b2	0.00142340	1.63789228
Prmt8	0.00149763	0.69218137
Hsd17b7	0.00158508	-0.92862897
Armh4	0.00162667	-0.69704755
Got1l1	0.00169645	2.02327625
Foxo6	0.00182587	0.89747095
Tmc4	0.00241028	0.61771605
Lmo1	0.00261155	0.59388295
Drc1	0.00266271	1.09400153
Cdh4	0.00293771	0.88816271
Mef2c	0.00299999	0.85976461
Akap5	0.00305353	0.71494285
Ecel1	0.00350562	0.89207296
Adora1	0.00372698	-0.58899960

SN – substantia nigra; MPTP - 1-methyl-4-phenyl-1,2,3,6-tetrahydropyridine; CBE - conduritol B epoxide; DEGs - differentially expressed genes

Table S3. Differentially expressed genes in SN from mice with MPTP+CBE compared to compared to mice with MPTP

DEGs	p-value	Log2FoldChange
Sgk1	$7.34*10^{-5}$	-1.39303692
Ucn	$3.72*10^{-4}$	5.10126015
Arl4d	$4.55*10^{-4}$	-1.06955067
Prmt8	0.00140147	0.69323273
Popdc3	0.00250220	0.83564281
Ankrd63	0.00258946	2.58085457
Pik3r6	0.00336224	1.20161688
Nkx6-1	0.00418106	0.90280976

SN – substantia nigra; MPTP - 1-methyl-4-phenyl-1,2,3,6-tetrahydropyridine; CBE - conduritol B epoxide; DEGs - differentially expressed genes

Table S4. Differentially expressed genes in SN from mice with MPTP compared to compared to mice with CBE

DEGs	p-value	Log2FoldChange
Parpbp	$7.15*10^{-9}$	7.75728016
Pmch	$1.32*10^{-8}$	6.89975245
Hcrt	$2.15*10^{-6}$	10.2272463
Ldb2	$4.73*10^{-5}$	0.80732283
Vangl1	$1.00*10^{-4}$	0.75320020
H2-Q2	$3.15*10^{-4}$	1.75304129
Syne4	$4.42*10^{-4}$	0.85847611
Tox	$4.56*10^{-4}$	0.94608529
Galr1	$7.17*10^{-4}$	1.98777749
Calcr	0.00123451	1.91549766
L3mbtl1	0.00178669	0.67146595
Nkd2	0.00180863	0.80701645
Tmem114	0.00217489	1.87679603
Slc24a2	0.00257441	-0.62294054
Prlhr	0.00287968	1.74111262
Gabre	0.00294519	1.37213337
Papln	0.00308649	0.99136525
Rrad	0.00386851	1.04071978
Adamts15	0.00417637	0.61223298
Gabrg3	0.00420713	0.78473896
Plk1	0.00421446	0.74063491
Ngb	0.00429757	0.93952362
Irx6	0.00440310	1.05900092

SN – substantia nigra; MPTP - 1-methyl-4-phenyl-1,2,3,6-tetrahydropyridine; CBE - conduritol B epoxide; DEGs - differentially expressed genes

Table S5. Differentially expressed genes in SN from mice with MPTP compared to vehicle mice

DEGs	p-value	Log2FoldChange
Marchf11	$6.12*10^{-6}$	-1.39441538
Ldb2	$6.97*10^{-6}$	0.92570483
Pmch	$1.08*10^{-5}$	4.90803528
Parpbp	$2.51*10^{-5}$	4.87574422
Prlhr	$4.25*10^{-5}$	4.16288499
Gm5535	$6.13*10^{-5}$	-2.07723070
Plagl2	$8.40*10^{-5}$	0.79668247
Il11ra1	$1.27*10^{-4}$	-0.73338837
Ttll3	$1.47*10^{-4}$	0.81263952
Hcrt	$3.27*10^{-4}$	5.42990022
Naa38	$3.52*10^{-4}$	-0.59561024
Zfp367	$3.89*10^{-4}$	-0.61555647
Hr	$4.01*10^{-4}$	0.90706627
Mybl1	$4.25*10^{-4}$	-1.10023703
Tox	$4.28*10^{-4}$	0.95559851
Galr1	$4.33*10^{-4}$	2.14210920
Islr2	$4.74*10^{-4}$	1.55262315
Rxrg	$4.75*10^{-4}$	1.44305501
Mreg	$5.17*10^{-4}$	-0.90997493
Pradc1	$5.29*10^{-4}$	-0.60925737
Aldh3b2	$6.37*10^{-4}$	1.86049836
Tead1	$6.46*10^{-4}$	0.60220973
Gck	$6.71*10^{-4}$	1.67727679
Gm5805	$7.07*10^{-4}$	-0.97674700
Npsr1	$8.36*10^{-4}$	1.60982807
Mgst3	$8.66*10^{-4}$	-0.86363937
Cast	$9.96*10^{-4}$	-0.73852800
Pdyn	0.00105191	1.84914286
Smc4	0.00123026	-0.72789888
Tbc1d4	0.00131115	-0.61766263
Gm10925	0.00140480	-0.64665629
Mapk15	0.00141277	2.70633496
Armh4	0.00149456	-0.71556109
Cox14	0.00154585	-0.59853593
Inhbb	0.00154993	-0.68488490
Cdh7	0.00156830	-0.67298463
Syne4	0.00175996	0.68369976
Kif6	0.00180070	0.86067353
Gabre	0.00185505	1.56838404
Kcna5	0.00200873	0.99216274
Cfap54	0.00201416	1.32978322
Tac2	0.00237660	2.69816659
Sv2b	0.00239419	-0.88481644
Apod	0.00250002	-0.81569282
Gabrg3	0.00258473	0.94540467
4933427D14Rik	0.00261159	0.91296547
Amigo2	0.00263749	0.74983049

Plxdc1	0.00268039	0.64568630
Parp3	0.00274272	-0.67492838
Gm17833	0.00286693	2.48960962
Rtl5	0.00291866	0.61842218
Myo18b	0.00297219	0.71620205
Hsd17b7	0.00300901	-0.79940140
Rskr	0.00308534	0.81083439
Ngb	0.00322447	1.11465781
Vamp8	0.00349565	-0.86815506
1700095J03Rik	0.00395746	1.20447328
Krt90	0.00400068	1.46304392
Rsph4a	0.00456301	1.20006114
Mycl	0.00458642	0.62218277
Crocc	0.00472544	0.60208596
Drc1	0.00486860	0.83598210
Fbxw7	0.00508217	-0.59395953
Itih3	0.00528863	0.65943353

SN – substantia nigra; MPTP - 1-methyl-4-phenyl-1,2,3,6-tetrahydropyridine; DEGs - differentially expressed genes

Table S6. Differentially expressed genes in SN from mice with CBE compared to mice with vehicle

**DEGs**	**p-value**	**Log2FoldChange**
Pomc	0.00027706	-2.64777546

SN – substantia nigra; CBE - conduritol B epoxide; DEGs - differentially expressed genes

Table S7. Venn diagram of DEGs in compared study groups

Groups	Number of DEGs	DEGs
Common		
MPTP+CBE vs CBE<br>MPTP+CBE vs MPTP<br>MPTP+CBE vs vehicle	2	*Ankrd63, Prmt8*
MPTP vs CBE<br>MPTP+CBE vs CBE<br>MPTP+CBE vs vehicle	1	*Slc24a2*
MPTP vs vehicle<br>MPTP+CBE vs CBE<br>MPTP+CBE vs vehicle	1	*Crocc*
MPTP vs CBE<br>MPTP vs vehicle<br>MPTP+CBE vs CB	1	*Syne4*
MPTP vs CBE<br>MPTP vs vehicle<br>MPTP+CBE vs vehicle	1	*Ldb2*
MPTP+CBE vs CBE<br>MPTP+CBE vs vehicle	3	*Zfp831, Neto2, Mef2c*
MPTP+CBE vs MPTP<br>MPTP+CBE vs vehicle	2	*Sgk1, Arl4d*
MPTP vs vehicle<br>MPTP+CBE vs vehicle	6	*Ttll3, Armh4, Hsd17b7, Islr2, Aldh3b2, Drc1*
MPTP vs CBE<br>MPTP vs vehicle	9	*Hcrt, Pmch, Gabre, Prlhr, Tox, Parpbp, Ngb, Gabrg3, Galr1*
Unique		
MPTP+CBE vs CBE	15	*Hecw1*, *Klhl11*, *Mirg*, *Dbn1*, *Ryr1*, *Pcgf2*, *Kif5b*, *Thbs3*, *Adam8*, *Cdk3*, *Atp13a5*, *Tnfrsf25*, *Arap2*, *Tbx2*, *Zc3h12c*
MPTP+CBE vs vehicle	21	*Adora1, Ddit4, Nsun7, Akap5, Rcor2, Foxo6, Tlcd4, Lmo1, Arrdc3, Pdk4, Npy2r, St18, Ciart, Idi1, Tiam1, Tmc4, Lrrn1, Rhpn2, Cdh4, Got1l1, Ecel1*
MPTP+CBE vs MPTP	4	*Popdc3 Nkx6-1 Pik3r6 Ucn*
MPTP vs vehicle	46	*Itih3*, *Mreg*, *Smc4*, *Mgst3*, *Cast*, *Npsr1*, *Cfap54*, *Marchf11*, *Gm5805*, *Zfp367*, *Gm5535*, *Sv2b*, *Gm10925*, *Mycl*, *Vamp8*, *Fbxw7*, *Kif6*, *Mybl1*, *Apod*, *Naa38*, *Plxdc1*, *Tbc1d4*, *Amigo2*, *Pdyn*, *Tead1*, *Gck*, *Inhbb*, *Rtl5*, *Mapk15*, *Rsph4a*, *Rskr*, *Hr*, *4933427D14Rik*, *Cox14*, *Pradc1*, *Myo18b*, *Plagl2*,

		1700095J03Rik, *Krt90*, *Il11ra1*, *Rxrg*, *Parp3*, *Gm17833*, *Kcna5*, *Tac2*, *Cdh7*
MPTP vs CBE	11	*H2-Q2, Vangl1, Tmem114, Plk1, Calcr, Nkd2, L3mbtl1, Adamts15, Papln, Irx6, Rrad*
CBE vs vehicle	1	*Pomc*

MPTP - 1-methyl-4-phenyl-1,2,3,6-tetrahydropyridine; CBE - conduritol B epoxide; DEGs - differentially expressed genes

Table S8. DEGs of SN of mice with MPTP+CBE vs vehicle mice involved in PI3K/AKT/mTOR pathway based on literature analysis

Genes	Number from list of DEGs	p-value	Log2FoldChange	Association with PI3K/AKT/mTOR pathway	References
Arl4d	1	$1.91*10^{-7}$	-1.8301	The expression of ARL4D was found to be post-transcriptionally regulated by the AKT/mTOR pathway and linked to the status of PTEN in human glioma.	[86]
Pdk4	2	$8.24*10^{-7}$	-1.0110	Knockdown of PDK4 suppressed the tumor development of cancer cells with activated mTORC1. The abundance of PDK4 dictated the responsiveness of cells to the mTOR inhibitor, rapamycin.	[58]
Sgk1	4	$5.53*10^{-6}$	-1.6660	mTOR, specifically mTORC2, is the hydrophobic motif-kinase for SGK1 Activated SGK1 contributes to the maintenance of residual mTORC1 activity through direct phosphorylation and inhibition of TSC2.	[57,87]
Ddit4	7	$8.26*10^{-5}$	-0.7205	DDiT4 is an inhibitor of mTORC1	[88]

Table S9. DEGs of PBMC-derived macrophages of GBA-PD (L444P/N) vs controls involved in PI3K/AKT/mTOR pathway based on literature

DEGs	Number from list of DEGs	p-value	Log2FoldChange	Association with PI3K/AKT/mTOR pathway	References
EGR1	3	$3.17*10^{-6}$	−4.1878	mTORC2 inhibition increases expression of EGR1	[89,90]

				Stimulation of mTORC1 increase expression of Egr1 regardless of its mRNA levels.	
DUSP1	4	$3.95*10^{-6}$	−3.2510	The activity of ULK1 is negatively regulated by MTOR, and inhibition of MTOR activates ULK, leading to autophagy induction. ULK1 phosphorylates BECN1 at Ser15 and promotes PIK3C3-BECN1-ATG14 complex formation and activation in dusp1−/− cells.	[91]
IL6	5	$3.95*10^{-6}$	−3.4155	IL-6 is a strong activator of mTOR	[92]
ARL4C	6	$5.06*10^{-6}$	−2.1475	AKT/mTOR inhibits ARL4C ubiquitation	[64]
NR4A2	7	$2.94*10^{-4}$	−3.0012	Nr4a2 Expression Is Induced by the PI3K-Akt-mTOR Pathway	[93]
